# New Insights on the Role of TRP Channels in Calcium Signalling and Immunomodulation: Review of Pathways and Implications for Clinical Practice

**DOI:** 10.1007/s12016-020-08824-3

**Published:** 2021-01-06

**Authors:** Saied Froghi, Charlotte R. Grant, Radhika Tandon, Alberto Quaglia, Brian Davidson, Barry Fuller

**Affiliations:** 1 Department of HPB & Liver Transplantation, Royal Free Hospital, Pond St, Hampstead, London, NW3 2QG UK; 2 Division of Surgery & Interventional Sciences/University College London (UCL), Royal Free Hospital, Pond Street, Hampstead, London, NW3 2QG UK; 3 HCA Senior Clinical Fellow (HPB & Liver Transplant), Wellington Hospital, St Johns Wood, London, UK; 4 Sheffield Medical School, Beech Hill Road, Sheffield, UK S10 2RX; 5 Department of Pathology, Royal Free Hospital, Pond Street, Hampstead, London, NW3 2QG UK

**Keywords:** TRP channels, Immunomodulation autoimmunity, Immunosuppression, Transient receptor potential channel

## Abstract

Calcium is the most abundant mineral in the human body and is central to many physiological processes, including immune system activation and maintenance. Studies continue to reveal the intricacies of calcium signalling within the immune system. Perhaps the most well-understood mechanism of calcium influx into cells is store-operated calcium entry (SOCE), which occurs via calcium release-activated channels (CRACs). SOCE is central to the activation of immune system cells; however, more recent studies have demonstrated the crucial role of other calcium channels, including transient receptor potential (TRP) channels. In this review, we describe the expression and function of TRP channels within the immune system and outline associations with murine models of disease and human conditions. Therefore, highlighting the importance of TRP channels in disease and reviewing potential. The TRP channel family is significant, and its members have a continually growing number of cellular processes. Within the immune system, TRP channels are involved in a diverse range of functions including T and B cell receptor signalling and activation, antigen presentation by dendritic cells, neutrophil and macrophage bactericidal activity, and mast cell degranulation. Not surprisingly, these channels have been linked to many pathological conditions such as inflammatory bowel disease, chronic fatigue syndrome and myalgic encephalomyelitis, atherosclerosis, hypertension and atopy.

## Introduction

**Fig. 1:**
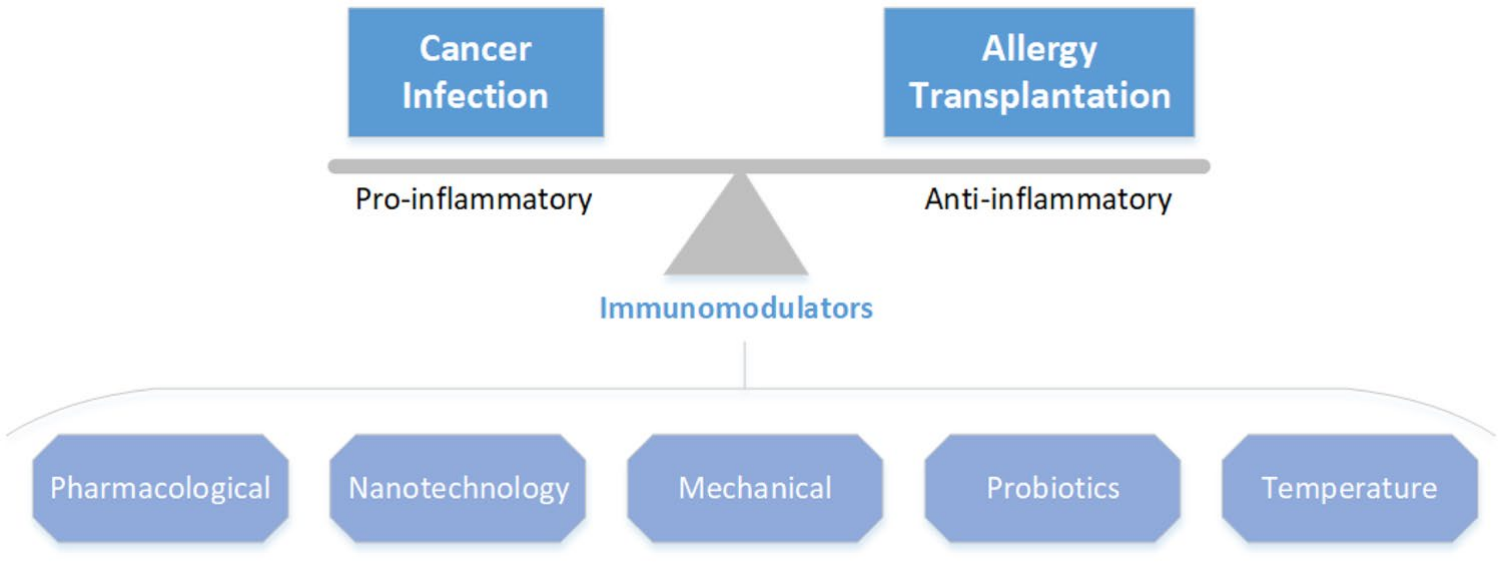
Immunomodulators can potentiate and aggravate pro- or anti-inflammatory responses based on the desired therapeutic end point. In addition to pharmacological strategies, other examples include alterations in materials used, mechanical stimulation. Biological modulation have been tried so far mostly for pro-inflammatory effects

Immunomodulation is the process which results in regulation or alteration of the scope, type, duration, or competency of an immune response [1]. The enforcers of such scheme, immunomodulators, can be both extrinsic or intrinsic. In its broadest sense, immunomodulation encompasses any intervention directed at modifying the immune response with a therapeutic end point. Such strategies have clinical importance in the development of new vaccines, treatment of autoimmune diseases and allergies, strategies in regenerative medicine, transplantation and immunotherapy for cancer (Fig. 1) [1–5]. Our understanding of the complexity of the immune system has changed greatly over the past decade which has resulted in trials of new therapies against cancer and a whole subset of other diseases. Central to this expansion is our better understanding of the molecular aspect of immune system machinery.

Calcium as the most abundant mineral in the human body plays an important role in the regulation of physiological processes and is also involved in many pathological disorders [6–9]. More so, it plays an important role in regulating immune function [10, 11]. There are many complex routes for calcium entry into the cell. Stimulation of immune cells results in the depletion of endoplasmic reticulum (ER) Ca^2+^ stores [12]. This seems to be sensed by stromal interaction molecule 1 (STIM1) located within the ER through interaction with plasma proteins, namely Orai1 protein. In turn, this results in sustained activation of calcium release-activated channels (CRACs) resulting in calcium influx, a process known as store-operated calcium entry (SOCE) (reviewed in [13]). Such sustained calcium influx across the cell membrane is important for lymphocyte activation and the initiation of both innate and adaptive immune response [11, 14, 15]. Other routes of calcium entry into the cell include voltage-gated calcium channels, IP3R cell surface receptors that are activated by IP3 ligand, P2X receptors and NMDA receptors [16]. This review focusses on transient receptor potential (TRP) channels, as they are widely expressed throughout the immune system, have varied roles and offer new therapeutic potentials.

**Fig. 2:**
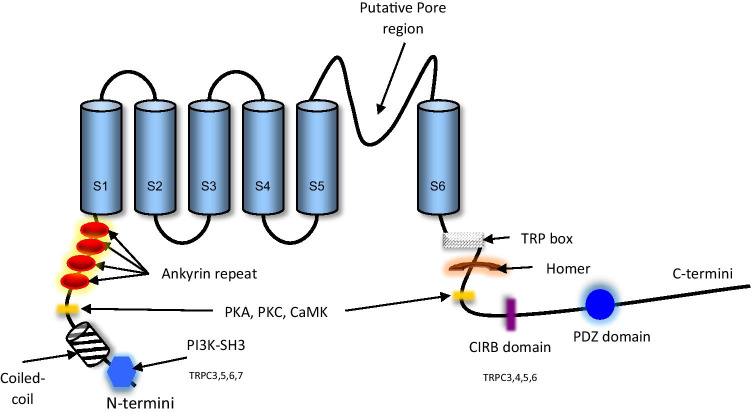
Structure of the TRP family with its 6 transmembrane (TM) domains spanning the cell membrane. Its putative pore region is located between S5 and S6 domains. The cytoplasmic portion of S6 helices form the lower gate, the opening and closing of which allows Ca^2+^ entry, hence regulating the flux of calcium into the cell. A functional channel requires assembly of 4 subunits. The N- and C-termini are preoccupied with several domains that regulate the function of the channel, i.e. activation. The ankyrin repeat domains can range between 0 and 14 in number. Normally seen in TRPA and TRPC subsets but absent in other members of the TRP family. TRP box is thought to be involved in gating mechanism and only present in TRPV, TRPC and TRPM subfamilies. CRIB, calmodulin/inositol-1,4,5-trisphosphate (Ins(1,4,5)P3) receptor binding domain; PDZ domain, postsynaptic density protein 95 (PSD95); PKA and PKC, protein kinase A and C; CaMK, calmodulin kinase

The TRP ion channels are a large and diverse family of proteins with their subunits united by a common primary structure and permeability to monovalent cations and divalent calcium ions (Fig. 2) [17–19]. They are involved in a continually growing number of cellular functions [20]. This is due to their large distribution in different organs. They have been found mainly not only in the brain but also in the heart, kidney, testis, lung, liver, spleen, ovary, intestine, prostate, placenta, uterus and vascular tissue [21]. They have also been found in many cell types, including both neuronal cells and non-neuronal tissues such as vascular endothelial cells, smooth muscle cells, as well as cells of the immune system [21]. In addition to being at the forefront of our sensor systems, responding to temperature, touch, pain, osmolarity, pheromones and taste [22, 23], they also play a role in vasorelaxation of blood vessels, metabolic stress and immune function regulation [21, 24]. Further to their physiological role, members of the TRP family are associated with several human diseases [25]. For example, mutations in the *PKD2* gene, which encodes the TRP polycystin 2 (TRPP2) protein, have been identified in autosomal dominant polycystic kidney disease [26]. The developmental disorder mucolipidosis is caused by mutations in the *MCOLN1* gene which encodes the TRP mucolipin 1 (TRPML1) channel [27]. Similarly, mutations within the TRP melastin 6 (TRPM6) channel are responsible for hereditary hypomagnesaemia and secondary hypocalcaemia [28]. There are less direct links to a range of autoimmune and inflammatory conditions such as asthma [29] and inflammatory bowel disease [30].

Therefore, a thorough understanding of TRP channels may enhance our knowledge of the underlying pathophysiology of an array of human conditions and potentially lead to novel therapeutic strategies.

After a brief introduction to TRP channel structure and function, the bulk of this review will cover evidence for TRP channel expression and function in individual immune cell populations. We will then highlight future approaches and new treatment options.

## Methodology

The literature search for this review occurred as the paper was being prepared between November 2017 and March 2018. Searches were performed using PubMed (NCBI, at https://www.ncbi.nlm.nih.gov/pubmed/). Search terms are included in Table 1. There were no formal inclusion or exclusion criteria but more recent publications written in the English language were favoured. The vast majority of studies were experimental rather than clinical.

**Table 1:** Search terms used to identify literature relevant to this review

Primary search terms	Additional search terms
TRP channel(s)	T lymphocyte or T cell
Transient receptor potential channel(s)	B lymphocyte(s) or B cell(s)
TRPA or TRP ankyrin	Natural killer cell(s) or NK cell(s)
TRPC or TRP canonical	Dendritic cell(s)
TRPM or TRP melastin	Macrophage(s)
TRPV or TRP vanilloid	Monocyte(s)
	Neutrophil(s)
	Mast cell(s)
	Platelet(s)

## Discussion

### TRP Channel Structure and Function

TRP channels are the most prominent emerging family of ion channels and the first to be identified in the post-genomic era using molecular biology approaches [31]. They are probably the most aggressively pursued drug targets over the past few years [32]. The revolution caused by sequencing the human genome substantially helped the identification of different members of TRP channels and facilitated an increase in the number of ‘players’ in many categories of biologically active proteins [20]. This is because, unlike other ion channels, TRP channels are identified by homology rather than by ligand function or selectivity due to their contrasting and unfamiliar functions [22]. Overall, they share 20–60% homology [20].

Based on sequence homology, the TRP family can be divided into three subfamilies: as short, long and osm-9-like [17] (a *C. elegans* TRP mutant) or TRPC (canonical) with seven members TRPC1–7; TRPV (named after the 1st group member vanilloid receptor) with six members TRPV1–6; TRPM (melastatin) has eight members TRPM1–8, in addition to TRPA (which has an ankyrin repeat domain). Other distantly related members of the mammalian family are TRPP (PKD) which lacks both ankyrin repeat and TRP domains [17, 21, 33–35]; TRPMN which lacks TRP domain and is characterised by its large ankyrin repeats domains; and TRPML subfamily (mucolipidin) where it plays a role in mucolipidosis type IV disease (developmental neurodegenerative disorder) [21]. These subfamilies are phylogenetically related [20] and TRPC family, which has been under considerable research especially with respect to their possible role in calcium entry [36, 37], is the most closely related member to the Drosophila TRP channel [20].

SOCE is a widespread phenomenon among cells where upon depletion of intracellular stores of calcium, cell surface channels are activated to allow entry of Ca^2+^ ions to replenish the stores. This highlights role of the Ca^2+^ entry for developmental and/or physiological process of different cells [38]. The first SOCE was identified by Hoth et al. [39] in mast cells and was named Ca^2+^ release-activated Ca^2+^ channels (CRAC, which is simply a specific SOCE with high Ca^2+^ selectivity; P_Ca_^2+^/Pa_Na_^+^ > 1000), and has been mainly found in haematopoietic cells [40, 41]. CRAC is subject to feedback inhibition by intracellular Ca^2+^ and is rightfully considered to be the best defined SOCE current that is activated by depletion of Ca^2+^ stores [40, 41]. Depletion of ER Ca^2+^ stores, in all non-excitable cells (apart from non-nucleated erythrocytes) and many types of excitable cells, causes activation of plasma membrane Ca^2+^-permeable channels [20]. This process is known as capacitative or store-operated Ca^2+^ entry, and the term ‘capacitative’ gives an appropriate impression as it is possible that close interactions between the ER and plasma membrane underlies SOC activation [20, 42, 43]. Above all, it should be noted that induction of *I*_CRAC_ does not necessarily require the depletion of stores and that other store depletion-stimulated currents or channels have been identified [44]. As previously alluded to, STIM1 have been shown to sense the depletion in calcium stores and result in sustained activation of CRAC via interaction with Orai1, which is an essential pore subunit of CRAC [45]. Mutations in Orai1 can result in immune deficiency by abolition of CRAC channel function [46].

### TRP Subfamilies in the Cells of the Adaptive and Innate Immune System

#### Cells of the Adaptive Immune System

##### T Lymphocytes

T cells comprise two main subsets. CD4^+^ T cells, or ‘helper’ T cells, are activated upon recognition of cognate antigen/major histocompatibility complex (MHC) class II which is expressed by specialised antigen-presenting cells. Antigen recognition leads to the production of a cocktail of soluble mediators such as cytokines and chemokines which orchestrate the subsequent immune response. CD8^+^ T cells, or ‘cytotoxic’ T cells, recognise antigen encased in MHC class I, which is expressed by the vast majority of human cell populations. Antigen recognition by CD8^+^ T cells triggers cytotoxicity. There are several other T lymphocyte populations and sub-populations (reviewed in [47]); however, the structure responsible for T cell activation in each case is the T cell receptor (TCR).

**Fig. 3:**
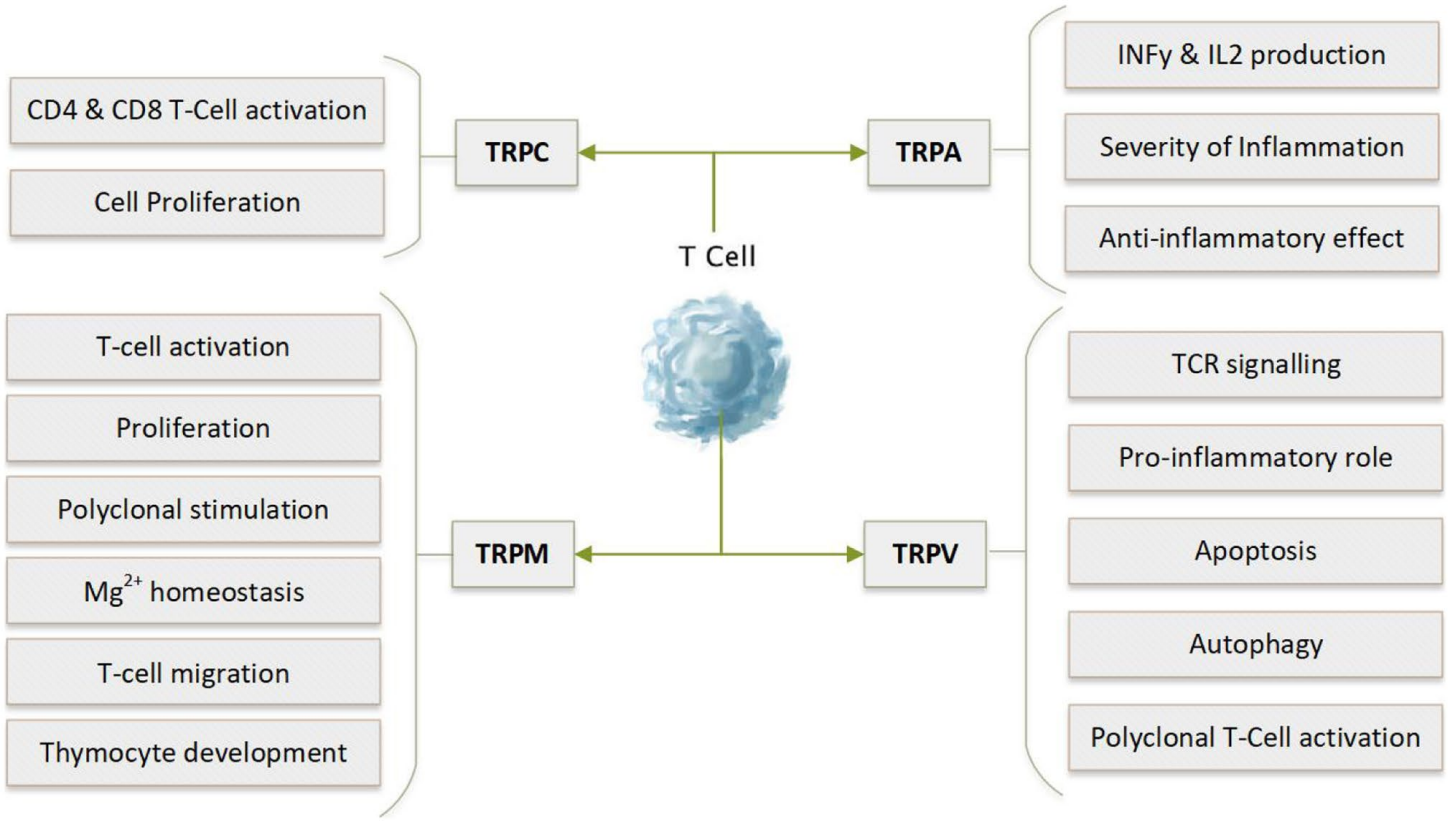
Widespread distribution of TRP channels and their role in the T cell

The details of TCR structure and signalling are complex and well beyond the scope of this review (reviewed in [14]). However, Ca^2+^ homeostasis is key to many downstream signalling pathways and effector functions. In summary, TCR stimulation leads to the recruitment of signalling molecules and adaptors to the TCR to form a proximal signalling complex. This results in the phosphorylation and activation of phospholipase-C (PLC)-γ, which cleaves phosphatidylinositol 4,5-bisphosphate (PIP_2_) to 1,4,5-inositol trisphosphate (IP_3_) and diacylglycerol (DAG). IP_3_ causes Ca^2+^ release from endoplasmic reticulum (ER) stores [14, 48]. Depletion of ER Ca^2+^ is sensed by STIM1 (stromal interaction molecule 1) whose Ca^2+^-binding domain is located on the luminal surface of the ER membrane [31]. STIM1 undergoes oligomerisation and translocation to the plasma membrane, enabling interaction with ORAI1 proteins, which are members of the CRAC channel [45]. This process, known as SOCE, is responsible for a large and sustained increase in intracellular Ca^2+^ levels [14, 48]. It is believed that several other Ca^2+^ channels expressed on the cell surface, including TRP channels, then modulate or fine-tune Ca^2+^ flux in T cells. Calcium signalling is crucial to the development and activation of T cells. Interestingly, STIM1 has been identified as a critical modulator of intracellular calcium in T cells [49]. Deficiencies of STIM1 expression have been linked to abnormal function of T cells. Here, we provide an overview of the role of TRP channels in the regulation of intracellular calcium in T lymphocytes (Fig. 3). The main TRP channels have been sub-grouped into TRPA, TRPC, TRPM and TRPV.

Identification and Function of the Individual TRP Channels in T Cells

TRPA

Stokes et al. [48] have demonstrated widespread expression of TRPA1 mRNA and protein throughout the human body, including in primary and secondary lymphoid organs such as thymus, spleen and lymph nodes [50]. They also demonstrated expression by Jurkat T cell lines [50]. Since then, the TRPA1 ion channel has been found to be expressed by both murine and human T cells [30].

TRPA1 has been linked to murine models of colitis and human inflammatory bowel disease (IBD) [30]. IL10^−/−^TRPA1^−/−^ mice developed more severe CD4^+^ T cell-medicated colonic inflammation than their IL10^−/−^ counterparts. In line with this, CD4^+^ T cells from TRPA1^−/−^ mice experienced a greater and more sustained level of calcium influx upon TCR stimulation compared to wild-type (WT) cells. This manifested in greater expression of the Th1 transcription factor Tbet and the Th1 cytokines IFNγ and IL2. Similarly, in human CD4^+^ T cells, TPRA1 knockdown increased IFNγ and IL2 production [30].

Bertin et al. [30] have also gone some way towards explaining how TRPA1 exerted these anti-inflammatory effects in their experimental systems. TRPA1 and TRPV1 were co-localised at the plasma membrane of CD4^+^ T cells, with TRPA1 inhibiting the activity of the TRPV1 channel. Reduced expression of TRPA1 promoted TRPV1-mediated TCR-induced calcium influx and CD4 T cell activation [30]. Interestingly, colonic biopsies from IBD patients have a greater number of infiltrating TRPA1^+^TPRV1^+^ leukocytes [51] and T cells [30] compared to controls. TRPV1 transcript levels are downregulated in biopsies from patients with IBD compared to controls [30], while the opposite pattern is true for TRPA1 [30, 51]. Bertin et al. [30] therefore suggest that differential expression of TRP channels or altered infiltration of TRPA1^+^TRPV1^+^ T cells contributes to the pathophysiology of this condition.

TRPC

A number of studies have demonstrated expression of TRPC channels by murine T cells [52–55], human T cell lines [56–60] and human T cells [56, 61].

Studies have suggested an immunosuppressive role for TRPC5, linking this channel to murine models of multiple sclerosis (MS) and type 1 diabetes (T1D). Wang et al. [52] demonstrated that activation of murine CD4 and CD8 T cells upregulated the expression of GM1, a ligand for galectin-1. Galectin-1 mediates suppression by regulatory T cells (Tregs), and in this study, it conferred protection against experimental autoimmune encephalomyelitis (EAE) [54]. CD4^+^ and CD8^+^ T cell activation also upregulated TRPC5, which led to Ca^2+^ influx upon GM1 cross-linking by galectin-1 [54]. Importantly, TRPC5 knockdown prevented galectin-1-mediated Ca^2+^ influx and Treg suppression [54]. In another study, effector T cells from non-obese diabetic (NOD) mice were found to express lower levels of GM1 compared to non-autoimmune prone strains. These T cells experienced lower Ca^2+^ influx via TRPC5 and were resistant to Treg suppression [55].

In contrast to the murine system, the expression of TRPC channels by human T cells has been a matter of debate. The use of a range of T cell lines probably explains this to some extent [62]. However, primary human CD4^+^ T cells consistently express TRPC1 and TRPC3 [56]. TRPC3 is upregulated upon polyclonal T cell stimulation, whereupon it promotes Ca^2+^-associated cell proliferation [56, 63].

TRPV

Studies have consistently demonstrated expression of TRPV1 and TRPV2 by human peripheral blood leukocytes/lymphocytes [64–66] and by CD4^+^ T cells [56, 67]. Expression of the other TRPV channels is more controversial [56, 64, 68], leading Wenning et al. [54] to question whether TRPV3-6 play a prominent role in human T cell biology [56]. For example, Wenning et al. [54] found TRPV6 expression by T cells from some human donors but not others, despite consistent expression by Jurkat T cell lines [56]. More recently, however, Majhi et al. [67] described expression of TRPV1-4 in resting Jurkat T cells and primary human T cells using confocal microscopy and flow cytometry [69].

TRPV1 plays a key role in TCR signalling. Upon TCR stimulation, TRPV1 co-localises with the TCR, and is phosphorylated by Lck [67], leading to Ca^2+^ influx [67, 69]. Consequently CD4^+^ T cells from TRPV1^−/−^ mice fail to produce pro-inflammatory cytokines after antigen-specific or polyclonal stimulation, and these T cells are unable to provoke colitis in IL10^−/−^ mice unlike their WT counterparts [67]. Moreover, treatment of human CD4^+^ T cells with TRPV1 antagonists or TRPV1 siRNA reduces the expression of activation markers and the production of IL2 [67]. As mentioned previously, TRPA1 regulates the activity of TRPV1 in CD4^+^ T cells, protecting against the development of T-cell mediated colitis in experimental models [30].

TRPV1 has a potential role in T cell development in the thymus. Subsets of murine thymocytes express TRPV1 [70, 71]. Exposure to capsaicin, the TRPV1 agonist, triggers apoptosis [71] or autophagy [70] via Ca^2+^ mobilisation. Capsaicin-mediated apoptosis has also been demonstrated in Jurkat T cells and activated, but not resting, human T cells [72]. In this study, however, the rise in intracellular calcium levels was shown to be independent of the TRPV1 channel [72].

TRPV2 is also expressed by human leukocytes/lymphocytes [64, 65] and CD4^+^ T cells [56]. In a patent application, Sauer and Jegla [71] describe accumulation of TRPV2 mRNA within CD4^+^ and CD8^+^ T cell populations, as well as an array of other immune system cells [73]. In the same patent application, the authors used shDNA to lower expression of TRPV2 in a bid to understand its mechanism of action. Knockdown of TRPV2 in Jurkat cells led to reduced TCR-induced calcium influx [73]. Jurkat T cells also express stretch-sensitive TRPV2 channels. Mechanical stress leads to Ca^2+^ influx, which, again, can be blocked by siRNA-mediated TRPV2 knockdown [74].

There are studies reporting expression of TRPV3 by murine and human T cells, although levels of expression are relatively low compared to other members of the TRPV family [52, 64, 67, 69]. Moreover, TRPV3^−/−^ mice have no discernible T cell phenotype [75].

Majhi et al. [67] demonstrated expression of TRPV4 by human T cells. Moreover, they show that exposure to a TRPV4 agonist triggers Ca^2+^ influx, and that exposure to a TRPV4 antagonist limits polyclonal T cell activation [69].

The role of TRPV5 and TRPV6 in T cell biology is more controversial [75]. Vassilieva et al. [74] demonstrated expression of both TRPV5 and TRPV6 by Jurkat T cells and human peripheral blood lymphocytes. Channel activation led to Ca^2+^ entry, which was blocked by a nonspecific inhibitor of TRPV5/6 channels [76]. However, TRPV6^−/−^ mice have no notable immunological defects or alterations in TCR-induced Ca^2+^ influx [75].

TRPM

TRPM2, TRMP4 and TRPM7 appear to be consistently expressed by human CD4^+^ T cells, and TRPM2 is upregulated strongly upon T cell stimulation [56, 62, 75].

TRPM2 channels play an important role in T cell activation via Ca^2+^ influx. And studies have begun to elucidate the mechanisms by which this occurs. For example, Ca^2+^ influx via TRPM2 can be stimulated by adenosine 5′-diphosphoribose (ADPR) and β-nicotinamide adenine dinucleotide (β-NAD), suggesting that these nucleotides act as second messengers [77, 78]. ADPR-induced Ca^2+^ influx can be triggered by oxidative stress induced by hydrogen peroxide [79] and by concanavalin A (conA) [80]. ADPR is believed to be endogenously generated by β-NAD hydrolysis [77, 81]. Indeed, the Ca^2+^ response to mitogens such as conA is limited by lowering NAD levels [81]. More recently, Fliegert et al. [80] have demonstrated that 2′-deoxy-ADPR is a more potent activator of TRPM2 than ADPR, labelling it as a ‘super-agonist’. Importantly, they show that 2′deoxy-NAD is present in Jurkat T cells [82].

CD4^+^ T cells from mice deficient in TRPM2 are less receptive to polyclonal stimulation, having reduced proliferation and cytokine-secreting capacity compared to those from WT mice [83]. TRPM2-deficient mice are also more prone to the development of EAE [83].

In contrast to other TRP channels, TRPM4 appears to dampen Ca^2+^ signalling in at least one subset of T cells [84, 85]. si-RNA-mediated reduction in TRPM4 by Jurkat T cells leads to prolonged Ca^2+^ influx and increased IL2 production upon activation [84]. Because TRPM4 is predominantly a Na^+^ channel, the proposed mechanism by which this occurs is via membrane depolarisation, and therefore a reduction in the driving force for Ca^2+^ entry via SOCE [84]. Weber et al. [83] have described important differences between Th1 and Th2 T cell subsets. Th2 cells express higher levels of TRPM4 compared to Th1 cells [83]. Furthermore, inhibition of TRPM4 in Th2 cells leads to increased Ca^2+^ influx, motility and production of IL2. The opposite was true for the Th1 subset [85].

The non-selective cation channel kinase TRPM7 is involved in cellular Mg^2+^ homeostasis [86]. Selective deletion of TRPM7 in developing thymocytes leads to developmental arrest at the double-negative (i.e. CD4^−^CD8^−^) stage [87]. The TRPM7^−/−^ T cells that do populate the periphery are unable to undergo apoptosis via the Fas-receptor pathway [88].

TRPM7 has also been linked to T cell migration. The presence TRPM7 at the uropod (the trailing edge of the cell as it migrates) is associated with Ca^2+^ oscillations required for migration. siRNA-mediated down-regulation of TRPM7 reduces the frequency of migrating cells and the speed of movement [89].

##### B Lymphocytes

**Fig. 4:**
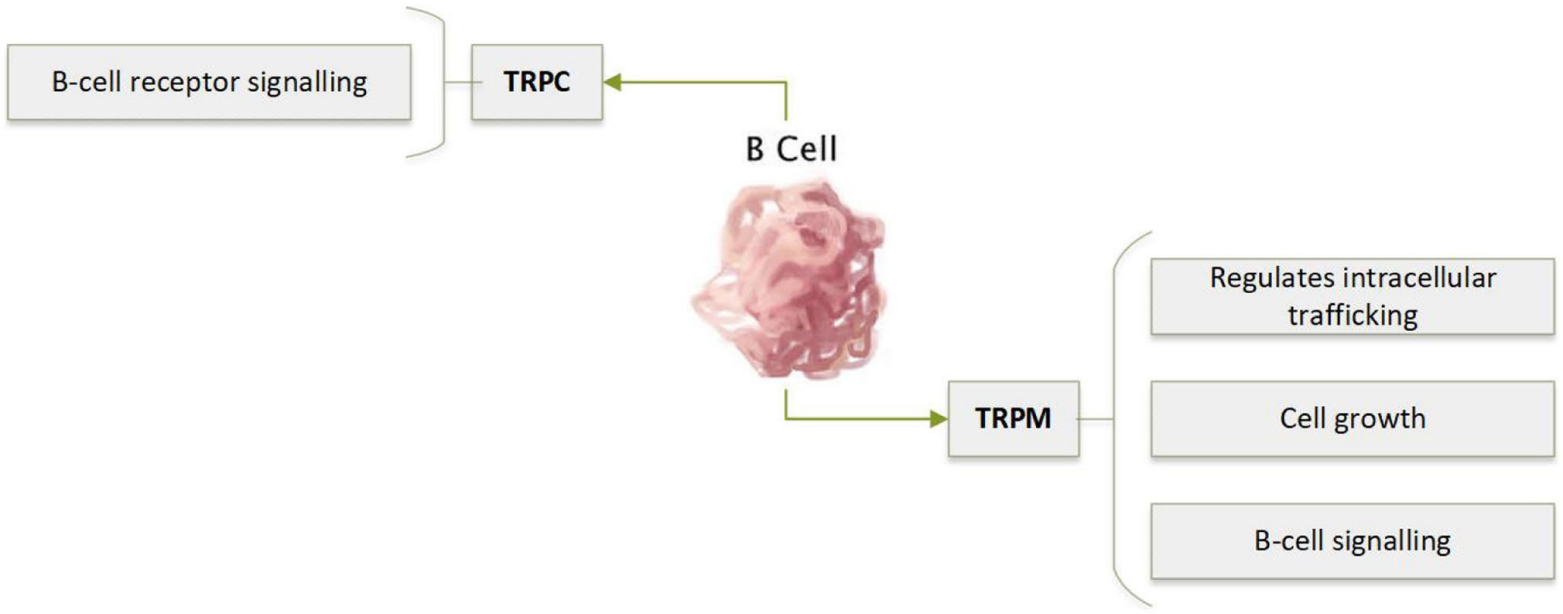
Expression of TRPC and TRPM in B cell and their role

B cells are responsible for antibody-driven immune responses. Like the TCR, activation of the B cell receptor (BCR) triggers Ca^2+^ influx via CRAC channels and the subsequent initiation of many adaptive immune functions (Fig. 4). Other Ca^2+^ channels, including TRP channels, have also been implicated in the regulation of Ca^2+^ influx in B cells (reviewed in [90]). Patients with mutations in the CRAC channel have a severe-combined immunodeficiency (SCID) phenotype. T cells from these patients display defective effector function; however, B cell activity appears to remain intact, suggesting that alternative Ca^2+^ influx mechanisms feature more heavily in B cells compared to T cells [91–93]. Despite this, our knowledge about the role of TRP channels in B cells is lacking.

Liu et al. [92, 152] demonstrated that primary human B cells express TRPC2, TRPC3 and TRPC6; TRPV2 and TRPC4; and TRPM1, TRPM5 and TRPM7 [94]. They also provided some evidence that one or more of these channels may be implicated in the B cell response to shear and osmotic stress; however, more work is required to elucidate the specifics of this signalling pathway [94].

Identification and Function of the Individual TRP Channels in B Cells

TRPC

Polyclonal CpG-mediated stimulation of B cells via the scavenger receptor B1 (SR-B1) leads to Ca^2+^ entry via PLCγ-mediated TRPC3 activation [95]. In BCR-induced signalling, it has been proposed that TRPC3 has dual roles. Firstly, TRPC3 is a DAG-activated Ca^2+^ channel, and secondly, it acts as a platform for PKCβ at the plasma membrane, thus promoting effective BCR signalling [96].

TRPC7 is involved in DAG-activated Ca^2+^ entry into DT40 B cells. TRPC7^−/−^ cells display impaired Ca^2+^ flux, which can be reversed by transfection with the human TRPV7 channel [97].

TRPM

Using DT40 B cells, Beulow et al. have shown that TRPM2 can be activated by oxidative stress, via poly(ADP-ribose) polymerases (PARPs) [98].

In DT40 B cells, TRPM6 kinase activity regulates intracellular trafficking of TRPM7 and controls TRPM7-induced cell growth [99]. TRPM7 phosphorylates PLCγ2, which is a key signalling molecule downstream of the BCR [100].

#### Cells of the Innate Immune System

##### Natural Killer Cells

Natural killer (NK) cells are involved in surveillance and protection against viral infection and malignant cell transformation. Their functions, such as cytotoxicity and cytokine production, are tightly controlled by a range of activating and inhibitory receptors expressed on the cell surface (reviewed in [101]). NK cells isolated from patients with an ORAI1 or STIM1 deficiency have defective SOCE, and they are unable to initiate effective cell killing due to impaired cytotoxic granule exocytosis [102]. This demonstrates the importance of SOCE to NK cell function. However, studies are beginning to reveal that other channels, including TRP channels, are involved in Ca^2+^ homeostasis in NK cells.

Identification and Function of the Individual TRP Channels in NK Cells

TRPC

**Fig. 5:**
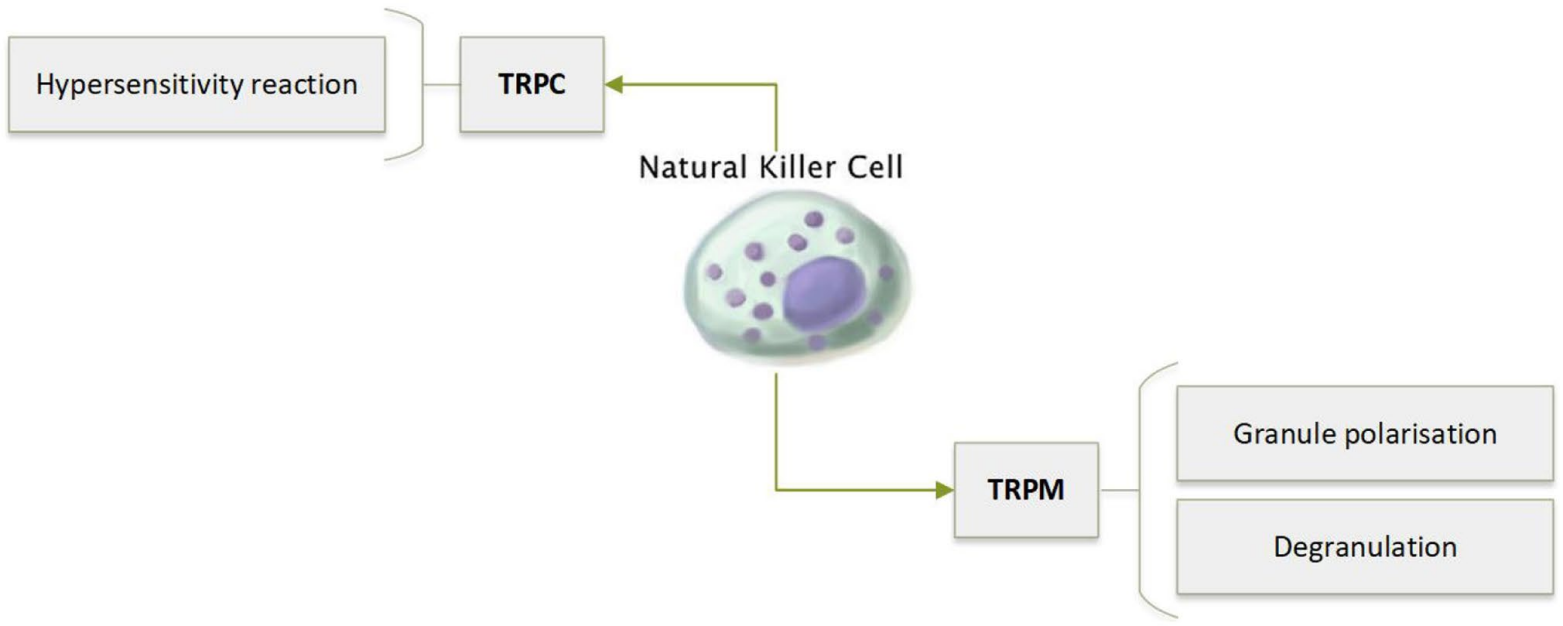
Role of TRP channels in regulating NK cell function via Ca^2+^ regulation

Recent studies have demonstrated that NK cells are able to directly respond to haptens. Stimulation with two haptens known to induce contact hypersensitivity cause Ca^2+^ influx, potentially via TRPC3 (Fig. 5) [103].

TRPM

The TRPM2 channel is involved in Ca^2+^ signalling within cytolytic NK cell granules. Upon recognition of a malignant cell, TRPM2 is activated by ADP-ribose, leading to Ca^2+^ mediated granule polarisation and degranulation [104].

TRPM3 is expressed on the NK cell surface [105, 106]. NK cells from patients with chronic fatigue syndrome/myalgic encephalomyelitis have lower expression of TRPM3, and altered cytoplasmic calcium flux, compared to those from healthy controls [105, 106]. Interestingly, Marshall-Gradisnik et al. [105] have discovered several single nucleotide polymorphisms associated with TRP ion channels (including TRPM3, TRPA1 and TRPC4) in patients with these conditions [107]. The role of NK cell functional defects in chronic fatigue syndrome and myalgic encephalomyelitis have yet to be fully explored.

##### Dendritic Cells

**Fig. 6:**
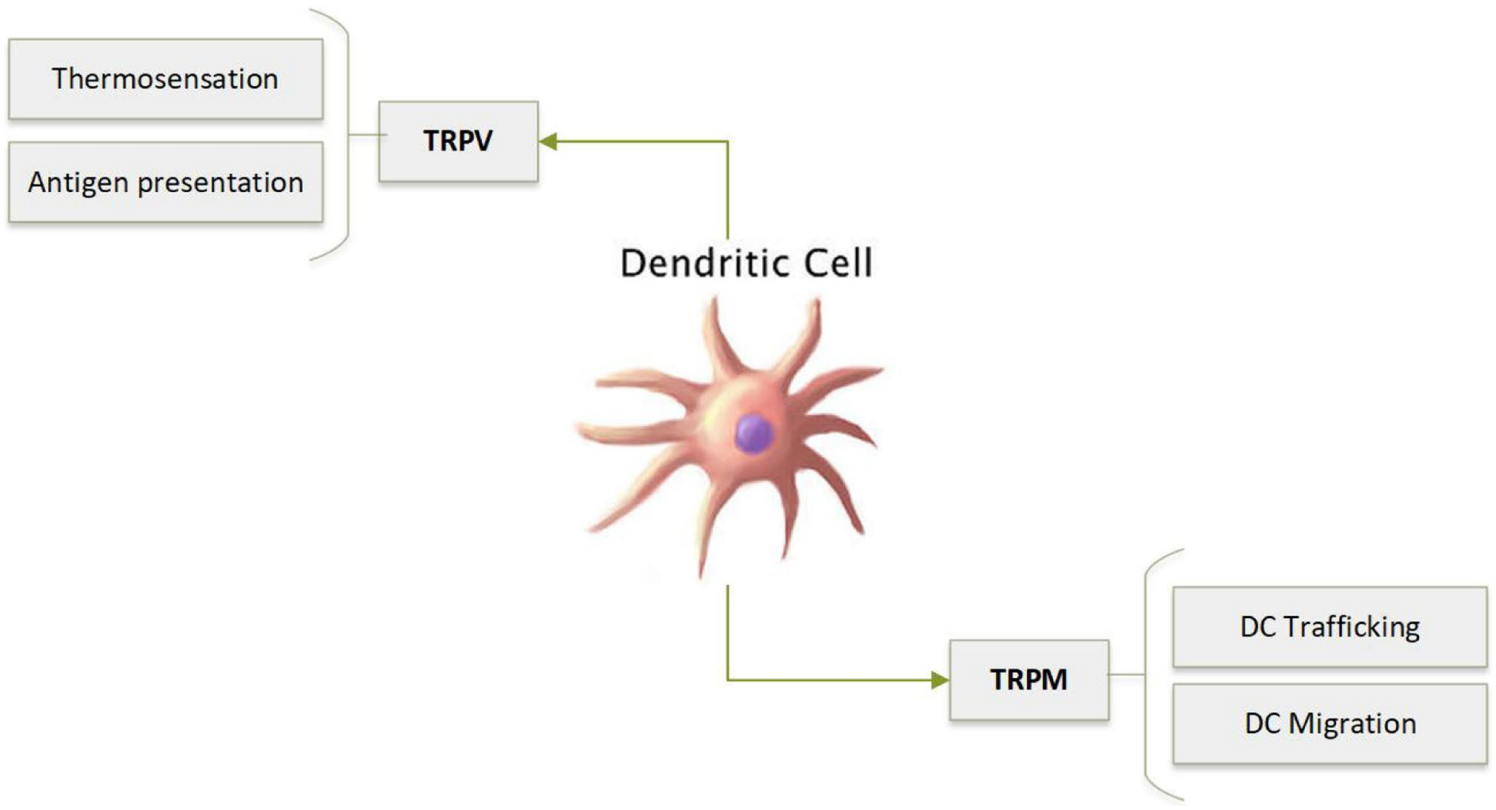
TRP channels in dendritic cells play a role in antigen presentation along with migration and trafficking

Dendritic cells (DCs) are the archetypal antigen-presenting cell. DCs recognise, process and display antigen encased in MHC class I or II to CD8^+^ and CD4^+^ T cells, thereby initiating an adaptive immune response. They are able to dictate the strength and direction of the immune response via cell surface receptors and the production of soluble mediators. Studies have described the CRAC channel as the predominant Ca^2+^ entry mechanism in DCs [108]; however, alternative Ca^2+^ entry pathways, for example TRP channels, are also believed to be involved in DC calcium flux (Fig. 6). Indeed, Vaeth et al. [107] demonstrated that SOCE via STIM1/2 is not required for many DC effector functions including phagocytosis, cytokine production and antigen presentation [109]. However, more recently, Maschalidi et al. [108] revealed a more prominent role for STIM1 in calcium regulation that is required for antigen cross-presentation and anti-tumour response [110]. STIM1 ablation leads to a decrease in cross-presentation. Interestingly, the endoplasmic reticulum Ca^2+^ sensor STIM1 seems to be activated by heat. Temperatures > 35 ℃ resulted in STIM1 clustering which then led to Orai-mediated Ca^2+^ influx as a heat off response [111]. Temperature seems to play an important role in immune system regulation and immune cell function [112–117]. One widely known benefit of fever is enhancement of immune response and release of pro-inflammatory cytokines that can further regulate immune cell function [116]. Furthermore, there is evidence for enhancement of DC function following fever, where elevated temperatures have substantially enhanced phagocytic ability of DCs [117–121]. The emerging evidence that intracellular calcium can be modulated to regulate immune function via STIM1 temperature-sensing mechanism would allow for potential tweaking of immune system function via pharmacological intervention or temperature.

Identification and Function of the Individual TRP Channels in Dendritic Cells

TRPV

Human DCs express TRPV1, TRPV2 and TRPV4 [122]. A role for TRPV1 in the generation of immune tolerance in the gut has been described. Ingestion of capsaicin promotes the development of tolerogenic antigen-presenting cells (APCs) in the murine lamina propria (LP) [123] or pancreatic draining lymph nodes [124]. DCs expressing the chemokine receptor CX3CR1 are believed to have regulatory properties [125]. In the LP, this population expresses higher levels of TRPV1 compared to their CX3CR1 negative counterparts [123]. Capsaicin is able to expand the size of regulatory antigen-presenting cells and enhance their tolerogenic properties, leading to protection from diabetes in NOD mice [123, 124].

Interestingly, TRPV1 may provide a link between the nervous and immune systems. After TRPV1, stimulation murine splenic DCs produce calcitonin-gene-related peptide (CGRP), a potent neuropeptide which has anti-inflammatory effects on both T cells and DCs [126].

TRPV2 has been linked to DC thermosensation. Temporary exposure to heat shock of 43 °C decreases endocytosis, an effect which can be reversed by TRPV2 siRNA [122].

TRPM

In bone marrow-derived DCs, TRPM2 expression is limited to endolysosomal storage vesicles, where it contributes to Ca^2+^ release upon stimulation with ADPR or chemokines [127]. DCs deficient in TRPM2 demonstrate impaired directional migration in response to chemokines or inflammatory mediators, suggesting that TRPM2 is involved in DC trafficking [127].

TRPM4 is also involved in DC migration, but via a different mechanism. Because TRPM4 is primarily a Na^+^ channel, blocking TRPM4 repolarises the plasma membrane, allowing for Ca^2+^ elevation [128]. This Ca^2+^ overload in TRPM4^−/−^ DCs impairs migration to lymph nodes in vivo [128].

##### Neutrophils

Neutrophils are keys to the initiation of an immune response. In the early stages of an infection, they perform phagocytosis, release toxic granules, oxidative bursts and neutrophil extracellular traps (reviewed in [129]). They also produce a cocktail of cytokines in order to help shape the direction of the ongoing immune response [130]. Regulation of these effector functions—in order to achieve an effective immune response while limiting host damage—occurs by careful sensing of environmental cues [131].

Following stimulation with, for example, chemoattractants, neutrophils experience a sustained increase in intracellular calcium via receptor-mediated calcium influx and SOCE [132]. The rise in Ca^2+^ concentration is essential for subsequent effector function [133–135]; however, similar to DCs, SOCE is dispensable [109], suggesting that other Ca^2+^ channels are heavily involved.

**Fig. 7:**
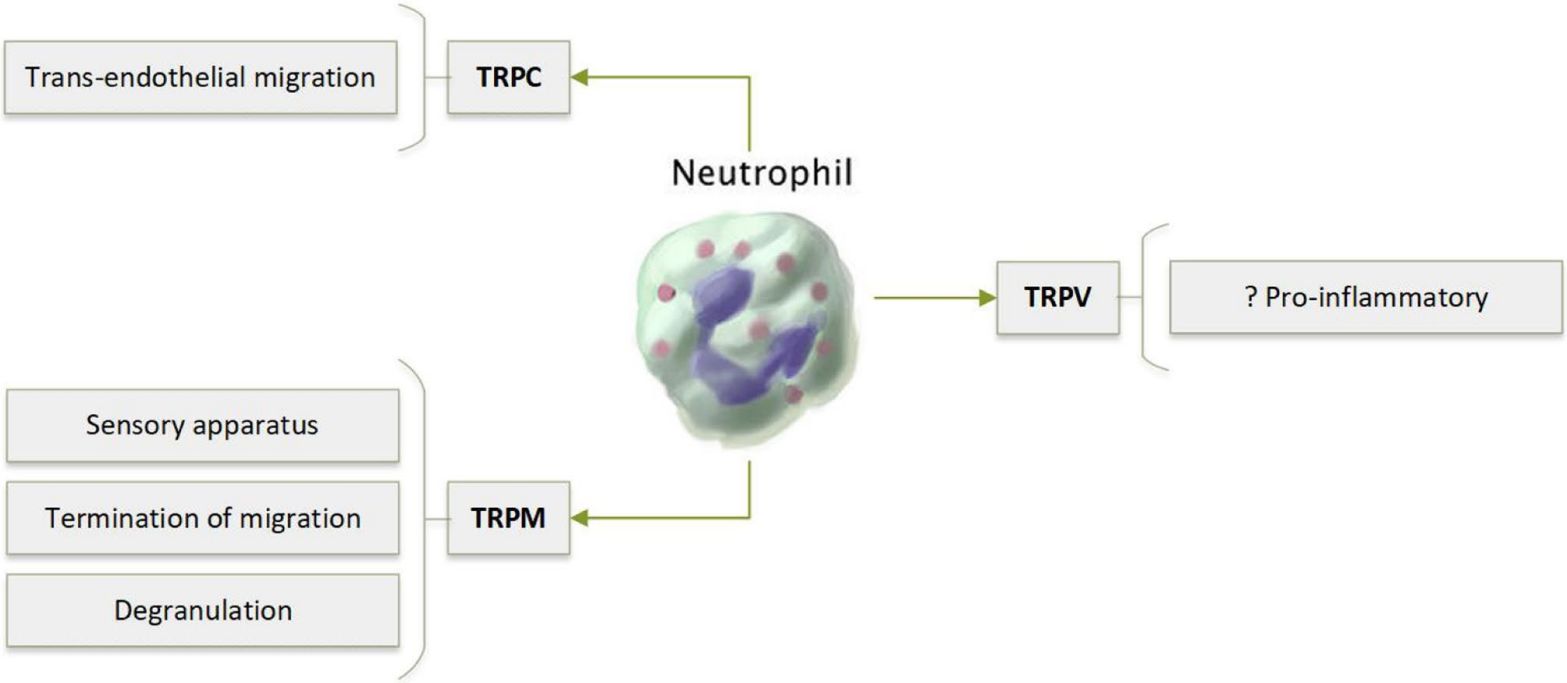
Calcium regulation in neutrophils via TRPC and TRPM channels both aids in initiation of trans-endothelial migration and its termination at the site of bacterial infection by sensing reactive oxygen species

TRPC, TRPV and TRPM channels have been linked to neutrophil function (Fig. 7).

Identification and Function of the Individual TRP Channels in Neutrophils

TRPC

Studies have demonstrated variable expression of TRPC1, TRPC3 and TRPC4, and consistent expression of TRPC6 by human neutrophils [136–138]. Bréchard et al. [137] demonstrated that TRPC3 acts primarily via SOCE-independent pathways whereas TRPC6 is involved in SOCE [139].

TRPC1 has been linked to the migration of murine neutrophils. TRPC1^−/−^ neutrophils have enhanced Ca^2+^ influx upon chemotactic stimulation, which leads to reduced migration and transendothelial recruitment [140].

Using TRPC6^−/−^ mice, Damann et al. [139] also demonstrated that TRPC6 is involved in neutrophil chemokine-induced migration [141]. TRPC6 was required for the correct organisation of filamentous actin in these migrating cells [141].

TRPV

Human neutrophils express TRPV1, TRPV2, TRPV4, TRPV5 and TRPV6 [136, 142]. There are, however, very few studies investigating the function of these channels.

Recently, Yin et al. [140] have shown that TRPV4 deficient neutrophils are less able to respond to proinflammatory stimuli in a murine model of acid-induced acute lung injury. However, the authors showed that TRPV4 expression by endothelial cells, rather than neutrophils, was more heavily involved in the pathophysiology of this condition [142].

TRPM

In contrast to DCs, TRPM2 is expressed on the plasma membrane of neutrophils, where it contributes to calcium influx [127, 136, 143]. TRPM2 forms an important part of the neutrophil sensory apparatus. By responding to environmental reactive oxygen species (ROS), TRPM2 terminates neutrophil migration at the site of infection, enabling for the appropriate initiation bactericidal activity [131]. Ca^2+^ influx via TRPM2 also primes neutrophil degranulation, increases proinflammatory cytokine production [144] and enhances bacterial killing [145]. In TRPM2^−/−^ mice with dextran sulfate sodium (DSS)-induced colitis, recruitment of neutrophils to the inflamed colon is impaired [146]. This reflects reduced production of chemoattractants by macrophages [146].

TRPM2 is detrimental in murine models of stroke, whereby it promotes cerebral inflammation due to the accumulation of neutrophils and macrophages [147].

##### Monocytes and Macrophages

Circulating monocytes and tissue-resident macrophages form a diverse group of phagocytic cell populations that help to orchestrate the immune response by producing chemokines and cytokines in response to microenvironmental signals (reviewed in [148]).

Similarly to neutrophils, intracellular Ca^2+^ fluctuations regulate many cellular functions in monocytes/macrophages, including phagocytosis [109, 149, 150].

**Fig. 8:**
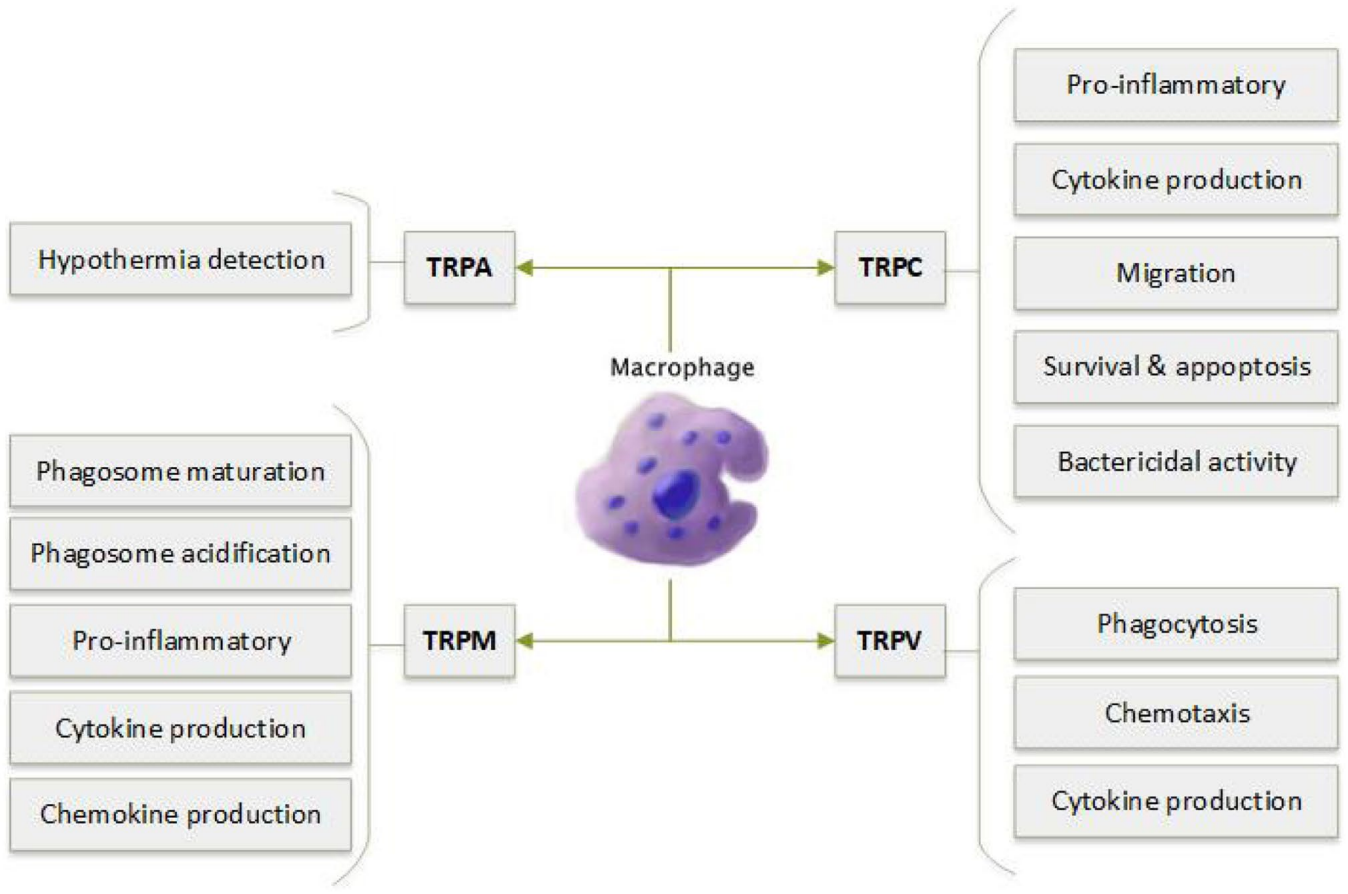
Varied role of TRP subtypes in the phagocytosis and pro-inflammatory cytokine production as well as migration of macrophages

The studies described below have linked expression of TRPA1; TRPV1 and TRPV2; TRPC1, TRPC3 and TRPC6; and TRPM2, TRPM4 and TRPM7 to monocyte/macrophage function (Fig. 8).

Identification and Function of the Individual TRP Channels in Monocytes and Macrophages

TRPA

Kun et al. [49] examined TRPA1 mRNA expression in colonic biopsies of mice with DSS-induced colitis and humans with inflammatory bowel disease. TRPA1 was found to be expressed by colonic macrophages [51]. Interestingly, inflamed colons had greater TRPA1 expression. Similarly, TRPA1^−/−^ mice had greater disease burden, suggesting that TRPA1 is protective in this condition [51]. However, the protective effect of TRPA1 in colitis has subsequently been linked to its role in T cells [30].

TRPA1 is responsible to monocyte detection of hypothermia, which has been associated with prolongation of inflammation in surgery [151].

More recent studies have suggested that TRPA1 protects against the development of atherosclerosis. Administration of a TRPA1 antagonist or genetic deletion of TRPA1 in apolipoprotein-E-deficient (Apo-E) mice increased plaque size, serum lipid levels and systemic inflammation [152]. Blocking TRPA1 expressed by macrophages led to lipid accumulation and the formation of foam cells in response to oxidised low-density lipoprotein (LDL) [152].

TRPC

TRPC1^−/−^ mice rapidly succumb to severe bacterial infection. Ca^2+^ entry via TRPC1 is required for TLR4-induced proinflammatory cytokine production by alveolar macrophages [153].

Elevated TRPC3 expression by monocytes has been linked to spontaneous hypertension in rats [154, 155]. Moreover, monocytes from human subjects with essential hypertension have higher levels of TRPC3 [156], which is associated with elevated proinflammatory cytokine production [157] and increased chemoattractant-induced migration [158]. Increased expression of TRPC3 and TRPC6 by human monocytes has also been associated with diabetes [159].

TRPC3 expression is linked with macrophage survival. TRPC3^−/−^ macrophages have increased rates of apoptosis, potentially due to reduced constitutive cation influx [160]. Using macrophages deficient in TRPC3, Solanki et al. [159] demonstrated reduced endoplasmic reticulum-stress induced apoptosis in the M1 inflammatory macrophage subset [161]. The same group later used the LDL receptor knockout mouse model of atherosclerosis transplanted with bone marrow from mice with a macrophage-specific loss of TRPC3, to further demonstrate that TRPC3-deficient macrophages have lower rates of apoptosis. The authors found a decreased number of apoptotic M1 macrophages and decreased plaque necrosis [162]. Recent RNA sequencing of M1 macrophages from mice with macrophage-specific TRPC deletion revealed 160 genes that were significantly differently expressed compared to their WT controls [163]. Genes that may be affected by loss of TRPC expression include those involved in cellular locomotion and lipid signalling [163]. Further work will need to be performed in order to fully understand the large amounts of data generated in this study.

Alveolar macrophages from chronic obstructive pulmonary disease (COPD) patients have increased TRPC6 expression, suggesting a potential role for TRPC6 in this condition [164].

Alveolar macrophages from cystic fibrosis (CF) patients have impaired acidification of phagosomes, which contributes to increased risk of chronic pulmonary infection. Riazanski et al. [163] have recently demonstrated that TRPC6 is able to restore macrophage bactericidal activity via translocation into the phagosomal membrane [165].

TRPV

TRPV2 plays a critical role in macrophage phagocytosis. The channel is required for phagocytic binding and internalisation [166]. TRPV2 is also involved in macrophage chemotaxis [167] and cytokine production [168]. Thus, TRPV2^−/−^ mice have impaired defence against bacterial infection [166].

Interestingly, after a myocardial infarction, TRPV2^+^ macrophages infiltrate the peri-infarct region [169]. TRPV2^−/−^ mice have a better recovery post-MI [170]. Moreover, infusion with WT macrophages leads to increased mortality, whereas this is not true TRPV2^−/−^ macrophages, suggesting that TPPV2 contributes to cardiac injury post-MI [170].

TRPM

TRPM2^−/−^ mice are more susceptible to infection compared to their WT littermates [171, 172]. Peritoneal macrophages from TRPM2^−/−^ have defective phagosome maturation, which prevents fusion of the phagosome and lysosome and stops effective clearance of bacterial pathogens [171]. TRPM2 is also essential for the appropriate acidification of macrophage phagosomes [173].

In addition to its role in phagocytosis, TRPM2 is also involved in lipopolysaccharide (LPS)-induced proinflammatory cytokine production [174], and in ROS-triggered chemokine production, and subsequent recruitment of neutrophils to the site of inflammation [146]. Thus, TRPM1^−/−^ mice are less susceptible to DSS-induced colonic ulceration [146].

Conversely, Di et al. [173] demonstrated a protective role for TRPM2 in endotoxin-induced lung injury, via inhibition of ROS production in phagocytes [175]. More recently, Beceiro et al. [174] have investigated the role of TRPM2 in Helicobacter pylori infection. They demonstrate that TRPM2 deficiency increases gastric inflammation and ROS production and reduces bacterial burden [176]. These conflicting results have led Beceiro et al. [174] to speculate that TRPM2 has distinct functions under different inflammatory conditions [176].

##### Mast Cells

**Fig. 9:**
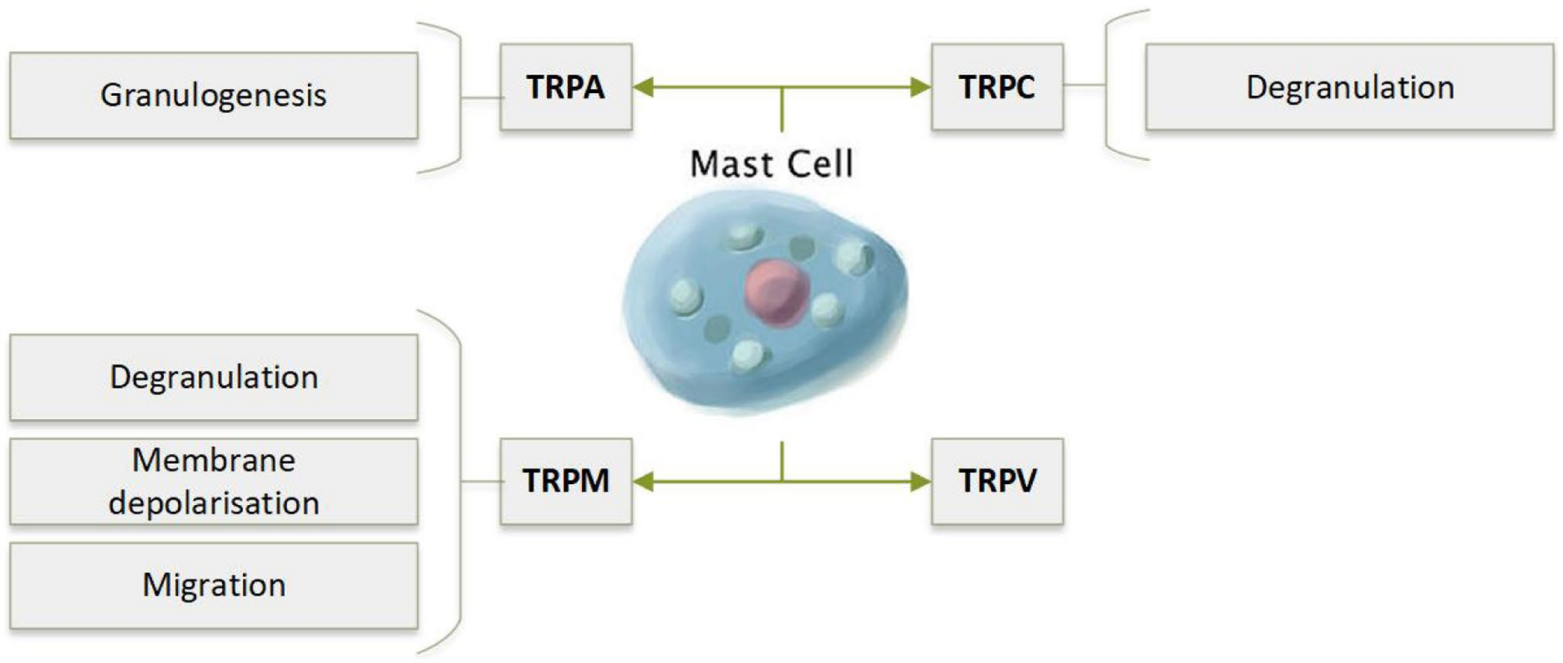
Calcium regulation via TRP channels in mast cells is essential for granulogensis and its release. TRPV is expressed in mast cells, but its functional significance is not clear

Mast cells are specialised to defend against external pathogens such as parasites, but they are perhaps most well known for their involvement in the pathophysiology of asthma and allergy. Mast cell activation leads to the release of preformed mediators such as histamine into the extracellular space, a phenomenon known as degranulation [177]. Calcium influx via CRAC channels is central to the formation of de novo inflammatory mediators, and for degranulation itself (reviewed in [178]). There is increasing evidence that TRP channels are also essential for mast cell effector functions (Fig. 9) [179].

Identification and Function of the Individual TRP Channels in Mast Cells

TRPA

TRPA1 has been detected in vesicular structures in the mast cell line RBL2H3, whereby it interacts with secretogranin III, a protein involved in granulogenesis [180].

TRPC

Studies in murine models have shown that TRPC1 [181, 182], TRPC3 [181] and TRPC5 [183] contribute to mast cell Ca^2+^ influx and/or degranulation. However, although Wajdner et al. [182] have recently described expression of TRPC 1 and TRPC6 by human mast cells, they found no evidence for a functional contribution of these channels to receptor-induced calcium entry [184].

TRPV

A recent study has demonstrated that, although TRPV1, TRPV2 and TRPV4 are expressed by murine peritoneal lavage mast cells, these channels are not mediators of Ca^2+^ elevation or degranulation in response to a variety of stimuli [185].

TRPM

TRPM2 is involved in allergen-induced degranulation in mast cells. Thus, antigen-stimulated degranulation and cytosolic Ca^2+^ concentration are reduced in TRPM2^−/−^ mice, and TRPM2 inhibitors reduce the pathological response to food allergens [186].

In contrast, TRPM4^−/−^ mast cells have elevated Ca^2+^ influx [187, 188] and release increased levels of histamine and other inflammatory mediators upon activation [187]. Moreover, TRPM4^−/−^ mice are prone to more severe anaphylactic responses after exposure to skin allergens compared to their WT littermates [187]. TRPM4 exerted this effect via membrane depolarisation, which limits the driving force for Ca^2+^ entry via the CRAC channel [187]. TRPM4 also regulates mast cell migration by regulating intracellular Ca^2+^ concentrations and modulating filamentus actin formation [189].

The TRPM7 channel is required for mast cell survival [190, 191] and function [29]. TRPM7 is expressed at higher levels in asthmatic rats compared to controls. Moreover, pharmacological blockade or shRNA-mediated knockdown of channel function reduced degranulation and cytokine release in asthmatic rats [29]. In contrast, Zierler et al. [190] found that TRPM7 kinase activity was able to regulate mast cell function independently of channel function by regulating the Ca^2+^ and Mg^2+^ sensitivity of G protein coupled receptor-mediated degranulation [192].

##### Platelets

Platelets are well known for their role in haemostasis, but increasing evidence demonstrates that they are keys to a coordinated and effective innate and adaptive immune response (reviewed in [193]). Ca^2+^ homeostasis is central to platelet function. Ca^2+^ levels are regulated by several mechanisms including SOCE via STIM1 and ORAI1 (reviewed in [194]). Human platelets express TRPC1, TRPC3, TRPC4, TRPC5 and TRPC6 [195, 196], as well as TRPV1 [197]. Increasing evidence suggests that TRPC channels are heavily involved in platelet activation.

Identification and Function of the Individual TRP Channels in Platelets

TRPC

The role of TRPC1 in platelets is somewhat controversial. SOCE can be inhibited in platelets by blocking TRPC1 function using a specific antibody [198] or reducing expression by shRNA [199]. However, TRPC1^−/−^ mice have intact SOCE [200]. The reason for this discrepancy remains unknown.

TRPC6^−/−^ mice have reduced or absent Ca^2+^ entry upon stimulation with 1-oleoyl-acetyl-sn-glycerol (OAG) [201, 202]. Studies have demonstrated involvement of TRPC6 in both SOCE [203] and SOCE-independent [204, 205] Ca^2+^ mobilisation. Although increasing evidence links TRP channel defects to cardiovascular and haemostatic pathology (reviewed in [206]), more work is required in order to understand associations with immunological or inflammatory conditions.

**Clinical Implications of TRP Dysfunction**

**Respiratory Pathology**

Acute respiratory distress syndrome (ARDS) is defined by acute hypoxemic respiratory failure, radiographic evidence of bilateral pulmonary opacities, and pulmonary oedema, not fully explained by cardiac failure or fluid overload [142]. It has an estimated incidence of 86.2 per 100,000 person-years and therefore presents a major cause of mortality and morbidity in critical care [142]. The cause can be due to numerous inflammatory triggers, both directly and indirectly including sepsis [142]. The characteristic pathology comprises of diffuse endothelial and epithelial injury, resulting in respiratory failure and formation of pulmonary oedema, and a strong inflammatory response characterised by the release of cytokines and the recruitment of granulocytes, monocytes and platelets into the lung [142].

TRPV4 has been implicated in this proinflammatory response and has been shown to be expressed on the key cell types involved in the pathogenesis of ARDS, as well as alveolar macrophages and neutrophils. TRPV4 also regulates the cellular responses in the pathogenesis of ARDS, such as lung endothelial barrier failure and macrophage activation. Studies have shown that therapeutic administration of TRPV4 inhibitors may alleviate lung injury in some, but not all, experimental models, and that further studies in different disease models and species are needed before this approach can be applied to patients [142].

COPD is currently sixth in the global impact of diseases and is predicted to be the third leading cause of death by 2020 [164]. A major risk factor for the development of COPD is cigarette smoking. Treatment for COPD is mainly symptomatic with no treatments that have any impact on the underlying inflammation of this disease. COPD encompasses chronic bronchitis, small airways disease and emphysema, with a characteristic feature of this disease being increased numbers of inflammatory cells located within the lungs of these patients. Studies have shown macrophages are increased numbers in the lung parenchyma of patients with COPD at the sites of alveolar destruction. These macrophages have been linked to TRPC6. Therefore, it has been suggested that these channels that might be responsible for much of the underlying pathophysiology of COPD [164].

##### Cardiology Pathology

Numerous TRP channels have been linked to cardiac pathology. Studies have shown up regulation of TRPV2 mRNA in the left ventricles (LVs) 3–5 days post-acute myocardial infarction and that TRPV2 expressing macrophages may play a significant role in the inflammatory processes that occur after permanent LAD occlusion at the local environment of the infarcted LV. TRPV2 gene overexpression may enhance the phagocytic activity of the peri-infarct macrophages [169].

In cardiac cells, several mutations in TRPM4 were found to be associated with human heart conduction dysfunction [35, 207]. Mutations have been also shown to be associated with progressive familial heart block type 1 [207].

Ischaemic stroke is the second most common cause of death worldwide. Studies have shown TRPM2 channels in neutrophils and macrophages regulate their migratory capacity to ischaemic brain thereby secondarily perpetuating brain injury. TRPM2-deficient mice are more protected from ischaemic stroke and show an improved neurological outcome compared with wild-type mice. TRPM2 activation in peripheral immune cells also leads to an exacerbation of ischaemic brain damage. Therefore, targeting TRPM2 systemically represents a promising therapeutic approach for ischaemic stroke [147].

The link between atherosclerosis, hypertension and TRPC3 has also been described in studies. The lack TRPC3 in macrophages was associated to an important reduction in plaque necrosis.[158, 162].

**Primary Hypomagnesemia with Secondary Hypocalcaemia**

Primary hypomagnesemia with secondary hypocalcaemia is an autosomal recessive disorder [28, 99]. It is caused by impaired intestinal absorption of magnesium accompanied by renal magnesium wasting due to a reabsorption defect in the distal convoluted tubule [28]. Mutations in the gene for TRPM6 were identified as the underlying genetic defect which included stop mutations, frame shift mutations, splice site mutations and deletions of exons [28]. This leads to patients failing to build a functional TRPM6 pore [99]. Patients with this disorder present in early infancy with neurological symptoms such as convulsions or muscle spasms.

Numerous studies have also established the role of magnesium as an essential nutrient contributing to the development of major risk factors leading to diseases, such as diabetes mellitus, hyperlipidaemia, atherosclerosis, and hypertension. This further highlights the importance of this nutrient and shows that TRPM6 genotyping or medications targeted to this area of the genome could be beneficial in the future.

**Autoimmunity**

Autoimmune diseases include diseases such as systemic lupus erythematous, rheumatoid arthritis, type 1 diabetes and multiple sclerosis. They occur when T cells attack a patient’s own cells [208]. T cell responses have also been implicated in graft rejection, allergy, asthma, dermatitis, psoriasis and graft versus host disease. Thus, treatment directed to inhibition of T cell activation and therefore TRP channels would be greatly desired to treat such undesired immune responses [73].

In addition to TRP cells in T cells, TRP channels have been show to be expressed in synoviocytes and studies have suggested that TRPV1-deficient mice develop reduced knee swelling [209].

**Allergy**

Activation and degranulation of mast cells is a key step in the pathogenesis of allergic diseases such as asthma and anaphylaxis [187]. An allergic reaction develops when allergens encountered by antigen-presenting cells are processed and presented to T cells. Studies have shown that TRPM4-deficient mice have a more severe acute anaphylactic response in the skin than control mice and therefore that TRPM4 channel activation is an efficient mechanism for limiting antigen-induced mast cell activation [10, 187].

Severe combined immunodeficiency syndrome (SCID) is a group of rare disorders caused by various mutations. Patients present with severe infections and usually die within the first few years of life. The standard treatment for SCID is stem cell transplantation or gene therapy [210]. TRPC channels have been linked to SCID and represent another potential treatment area [48].

**Chronic Fatigue Syndrome**

Chronic fatigue syndrome, also referred to as myalgic encephalomyelitis (ME) is a disorder identified by unexplained, debilitating fatigue accompanied by other neurological, immunological, autonomic and ion transport impairments [105, 106]. It has an unknown aetiology, and there are no specific diagnostic tests [107].

The most common finding reported in ME has been reduced NK cell cytotoxic activity [106, 107]. Atypical single nucleotide polymorphisms of the TRPM3 gene, from peripheral blood mononuclear cells, NK and B cells have been recently reported in ME groups compared with healthy controls [105, 106]. In addition, studies have shown a significantly reduced expression of TRPM3 on NK and B lymphocytes in ME patients [105]. This results in changes in Ca^2+^ ion concentration in the cytosol and intracellular stores which may change the NK cells’ activation threshold [106].

**Polycystic Kidney Disease**

TRPP subfamily has been linked to numerous cases of polycystic kidney disease [21, 207]. The proteins involved, PKD2, PKD2L1 and PKD2L2, are Ca^2+^ permanent channels called TRPP2, TRPP3 and TRPP5, respectively. Autosomal dominant polycystic kidney disease is caused by mutations in TRPP1 or TRPP2 which leads to alterations in the polarisation and function of cyst lining epithelial cells. Studies have shown that Mice with negative TRPP1 or TRPP2 are more likely to die in utero with cardiac septal defects and cystic changes in nephrons and pancreatic ducts [22].

##### Mucolipidosis

Mucolipin-1 (MCOLN1) is a novel membrane protein that is defective in mucolipidosis type 4 disease. It is part of the TRPML1 subfamily. Mucolipidosis is a developmental neurodegenerative disorder characterised by a lysosomal storage disorder, abnormal endocytosis of lipids and accumulation of large vesicles [21, 27]. Symptoms include severe psychomotor developmental delay, progressive visual impairment and achlorydia. It is an autosomal recessive disease, which typically presents in infancy. After the disease onset, a period of stability often ensures lasting for two to three decades. Treatment for the disorder includes enzyme replacement therapy, substrate reduction therapy and gene therapy [27, 207].

##### Inflammotory Bowel Disease IBD

TRPV1 has been linked to a number of conditions including inflammatory bowel disease [16, 67, 208].

TRPV4 may also have a role in colonic afferents as it is expressed in nerve fibres of patients with inflammatory bowel disease [23]. Inflammatory bowel diseases include ulcerative colitis and Crohn’s disease. Ulcerative colitis affects only the colon, Crohn’s may affect all parts of the gastrointestinal tract, but most commonly the distal part of the small intestine, the ileum and the colon. Clinical symptoms of IBD comprise of abdominal pain, diarrhoea, gastrointestinal bleeding and weight loss. The main management of IBD is immunosuppressive therapies, which are associated with significant adverse effects or it often has no effect on the disease [51].

Crohn’s is characterised by a T helper 1-mediated inflammatory response with overproduction of interferon and tumour necrosis factor. Ulcerative colitis is a T helper cell 2-mediated immune disease with massive production of interleukins 5, 9 and 13. As previously discussed, TRPV1 channels are expressed in CD4 T cells which increases their pro inflammatory properties in models of colitis. Studies have also shown mice without the TRPA1 develop severe spontaneous colitis [30].

##### Oncology

There are many neoplasms associated with TRP changed. TRPM1 is linked to the production of malastatin. This gene expression correlates with cutaneous melanoma tumour progression, thickness and potential for metastasis in normal skin, benign melanocytes naevi (moles) and primary cutaneous melanoma metastasis. Loss of the TRPM1 mRNA in the primary cutaneous tumour has been proven as a marker for metastasis in patients with melanoma [17, 21, 207].

TRPV2 overexpression was evidenced in patients with multiple myeloma. In hepatocellular carcinoma, it was associated with medium and well-differentiated tumours, where it was proposed as a prognostic marker. In prostate cancer, TRPV2 was shown to be involved in cancer cell migration and invasion, and may be specifically implicated in the progression to more aggressive phenotype. On the other hand, TRPV2 was shown to negatively control proliferation and resistance to Fas-induced apoptosis of glioblastoma multiforme [74].

TRP channels are heavily involved in calcium and vitamin D signalling in breast cancer. TRPV6 has been shown to be upregulated up to 15 times in breast cancer tissue when compared with that in normal breast tissue. The expression level of TRPV6 is also reduced in breast cancer cell lines in the presence of tamoxifen, an antagonist of oestrogen [6]. TRPV6 expression is also upregulated in prostate cancer and other cancers of epithelial origin, highlighting its potential as a target for cancer therapy [76].

Human myeloid leukaemia cells coexpress functional TRPV5 and TRPV6 calcium channels. Levels of both TRP channel have been found to be significantly higher in malignant cells than in quiescent lymphocytes. This indicates that TRPV6 upregulation is associated with increased proliferative activity in leukaemic cells and in activated lymphocytes which is in agreement with data showing elevated expression of TRPV6 in colon, breast, thyroid, ovarian and pancreatic carcinomas in comparison with normal tissues [76].

There are also numerous other TRP channels associated with cancers including TRPM4, which is linked to a variety of childhood and adult tumours and the cancer predisposing Beckwith-Wiedemann syndrome [17]. Downregulation of TRPC6 has been shown to be associated with autocrine tumours [21]. An exon 9 deletion in TRPC1 has also been linked to human ovarian adenocarcinoma [207].

##### TRP Pharmaco-immunomodulation (Summarised in Table 2)

Recent studies highlighting novel associations between TRP channels and the immune system point at potential drug targets for the future.

**Table 2:** Most commonly used TRP channel pharmacology

TRP channel target	Name of drug	Pathology targeted	Action	Possible side effects	Development status (experimental or available)
TRPV1	Capsaicin	Neuropathic and nociceptive pain	Analgesic	Altered body temperature, reduction in stimuli response	Available
TRPV1	Resiniferatoxin	Ablate nociceptor	Palliative pain	None	Under phase 1 trials
TRPV1	Piperidine, euginol, gingerol, anandamide	Vasolidation		Unknown	Under phase 1 trials
TRPV2	Cannabidiol	Bladder cancer cells	Induces apoptosis	Liver damage, sedation and mood changes	Available

**Fig. 10:**
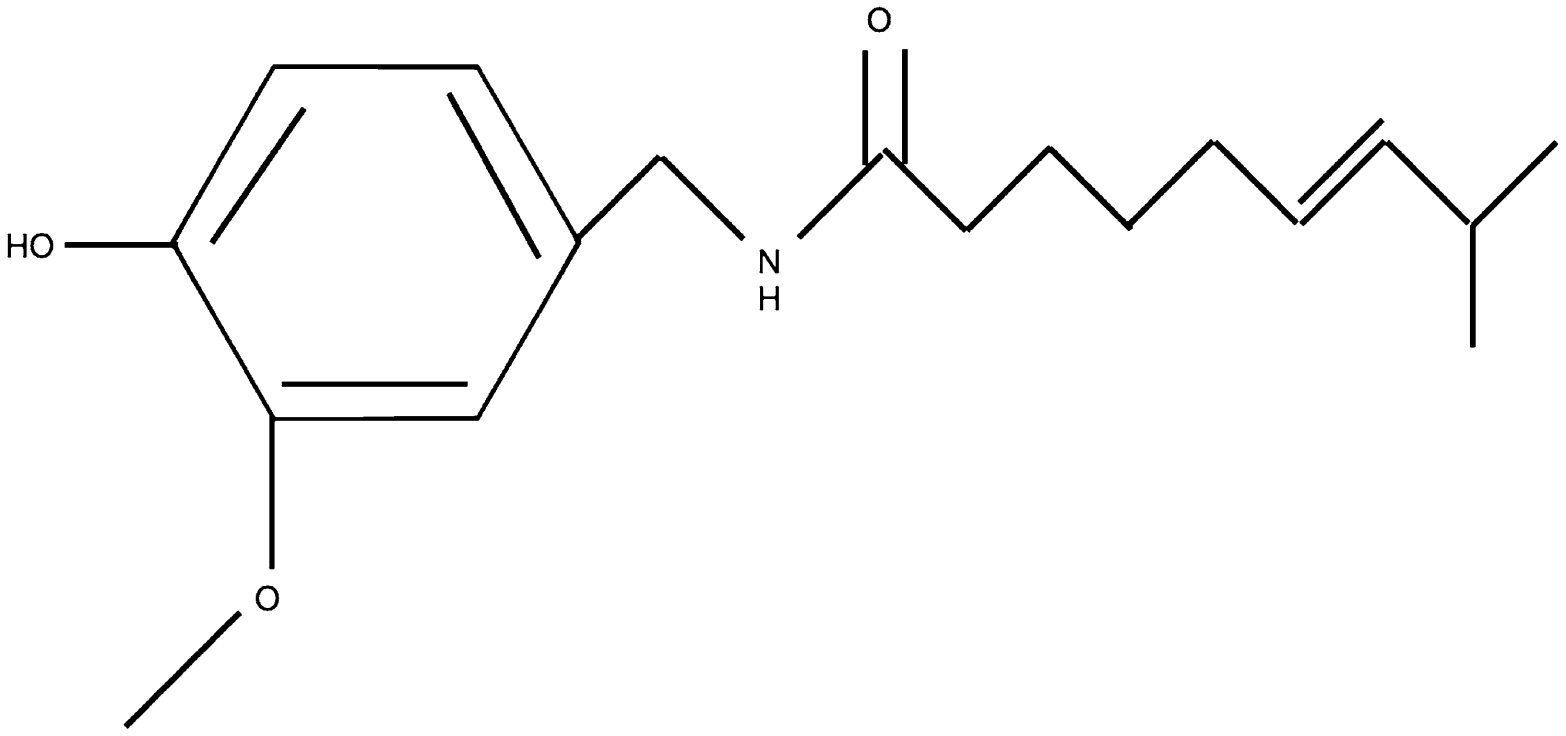
The chemical structure of capsaicin [23]

TRPV1 activators, for example capsaicin and resiniferatoxin (RTX), are among the most well-known TRP channel pharmacology (Fig. 10).

Capsaicin activates TRPV1 which can enhance regulatory macrophages in the gut [123]. It can also inhibit prostaglandin production in macrophages. They can lead to a large calcium influx that can produce degeneration of nociceptor axons at the site of applications into joints or onto nerves. They may even cause a loss of the sensory neuron itself by calcium-mediated mitochondrial damage and cytochrome c release leading to apoptosis when exposed close to the cell body [23]. It has been shown pharmalogically to induce a number of effects in different cell types including cell death [70].

Capsaicin can be used experimentally as an analgesic agent in the treatment of painful disorders such as peripheral neuropathy and rheumatoid arthritis [72]. Other uses include detrouser hyperreflexia, interstitial cystitis, pruritus associated with chronic renal failure and diabetic neuropathy [65]. When used in addition to a derivative of lidocarine, much more long-lasting pain relief can occur without impairing motor function or tactile sensitivity [82]. Capsaicin also exhibits anti‐inflammatory properties [69]. This is also known as Neuroges X when used for neuropathic post surgical pain.

Although studies have demonstrated that this molecule can promote immune tolerance in a murine model of type 1 diabetes [123, 124], future studies should examine its effects on TRP channels in human autoimmune conditions. There are also concerns about using this drug pharmacologically for side effects such as diminished response to damaging heat stimuli, altered body temperature and a reduction in the perception of taste which need to be fully explored before use on patients [23].

Resiniferatoxin is a more potent analgesic than capsaicin and can selectively ablate nociceptors when delivered intrathecally, which may have special utility for uncontrolled pain in a palliative setting [82]. This drug is currently under phase 1 trials and is also a TRPV1 agonist [208, 209]. It can be delivered by injection into the subarachnoid space in the spinal cord or into areas of the skin where nerves terminate [209]. Trials have suggested that the drug can be used for pain relief in numerous pathologies including Morton’s neuroma, neuropathic pain and burns. There are minimal side effects with its use [209].

Other exogenous TRPV1 agonists include piperidine, eugenol, gingerol and anandamide, as well as noxious heat (> 43–45 °C). These are still under trials, and side effects and uses are largely unknown. Anadamide is an endogenous cannabinoid receptor agonist that can also be used to induce vasodilation by activating vanillin receptor on perivascular sensory neurons [21, 126].

The main pharmacological tool for TRPV2 is cannabidiol, the major non-psychotropic cannabinoid compound derived from plant *Cannabis sativa*. It is a relatively selective TRPV2 agonist. It has been shown that administration of cannabidiol was shown to induce apoptosis in human T24 bladder cancer cells due to continuous influx of Ca^2+^ through TRPV2, and proposed as a potential therapeutic target for human urothelial carcinoma [74]. There are 2 main approved drugs available that use this compound (dronabinol and nabilone). There are also numerous side effects including liver damage, sedation and mood changes [210].

There are also a number of other pharmacological agents currently in trials with little information known about them:

1. Adenosine 5′-diphosphoribose (ADPR) has been shown to be the main agonist for TRPM2 [82]. This has been used in experimental trials
2. The TRPV1 antagonist, SB-705498 is currently the only published TRPV1 clinical study with efficacy data. It has been shown to reduce the area of capsaicin-evoked flare and to increase pain tolerance at the site of ultraviolet B irradiation [82].
3. TRPM2 inhibitors include clotrimazole, econazole and flufenamic acid [144].
4. HC-030031 is so far not used clinically but has been orally used in rat models and appears to be safe. It has been shown to be used as treatment options for hypothermia or instead of capsaicin [151].
5. TRPC3/6 selective antagonist (GSK-3503A) and agonist (GSK 2934A) are in trials [184].
6. A TRPV4 antagonist, GSK2220691, is not currently available, but there is also a commercially available TRPV4 inhibitor, HC-067047 [142].
7. Ruthenium red is a non‐selective TRP channel blocker, can suppress lipopolysaccharide (LPS)-induced tumour necrosis factor α (TNFα) and interleukin‐6 (IL‐6) production in macrophage cells [69].
8. Naturally occurring cinnamaldehyde is a TRPA1 agonist, which induces a vasorelaxant action via endothelium-dependent or endothelium-independent mechanisms [152].

Recent studies identifying these more potent TRP agonists [82] highlight how little we know about the intricacies of TRP channel activation and signalling. Studies should continue to elucidate these mechanisms in order to reveal important avenues for research into the pathophysiology of human disease and identify more potential therapeutic targets. Recent studies highlighting novel associations between TRP channels and the immune system point at potential drug targets for the future. Future studies should also examine its effects on TRP channels in human autoimmune conditions, as there is a lack of potential pharmacology in this area.

## Conclusion

TRP channels have emerged as an essential component of calcium signalling machinery. TRP channels are involved in the activation of both innate and adaptive immune system cells. With links to diverse pathological conditions, including autoimmune and inflammatory states, TRP channels represent a promising future therapeutic target. The fact that these channels can sense changes in pH, temperature or even mechanical stress and change the function of the cell leaves these receptors amenable to a wide range of modulators. This leaves a window of opportunity to modulate immune cells via different means.
